# Overview of the Semiconductor Photocathode Research in China

**DOI:** 10.3390/mi12111376

**Published:** 2021-11-09

**Authors:** Huamu Xie

**Affiliations:** State Key Laboratory of Nuclear Physics and Technology, Institute of Heavy Ion Physics, Peking University, Beijing 100871, China; hmxie@pku.edu.cn

**Keywords:** photocathode, accelerator, electron gun, electron source

## Abstract

With the growing demand from scientific projects such as the X-ray free electron laser (XFEL), ultrafast electron diffraction/microscopy (UED/UEM) and electron ion collider (EIC), the semiconductor photocathode, which is a key technique for a high brightness electron source, has been widely studied in China. Several fabrication systems have been designed and constructed in different institutes and the vacuum of most systems is in the low 10^−8^ Pa level to grow a high QE and long lifetime photocathode. The QE, dark lifetime/bunch lifetime, spectral response and QE map of photocathodes with different kinds of materials, such as bialkali (K_2_CsSb, K_2_NaSb, etc.), Cs_2_Te and GaAs, have been investigated. These photocathodes will be used to deliver electron beams in a high voltage DC gun, a normal conducting RF gun, and an SRF gun. The emission physics of the semiconductor photocathode and intrinsic emittance reduction are also studied.

## 1. Introduction

Many scientific projects such as a high repetition rate XFEL [1,2,3], electron-ion collider (EIC) [4], ultrafast electron diffraction/microscopy (UED/UEM) [5,6,7], etc., are being built or proposed in China. In these big projects, the demand for a high-performance photocathode has become more crucial and urgent, especially for a semiconductor photocathode [8]. For instance, a Cs_2_Te photocathode is the first choice of a hard XFEL application such as a **S**hanghai **HI**gh repetitio**N** rate XFEL and **E**xtreme light facility (SHINE) and a Shenzhen Superconducting Soft X-Ray Free Electron Laser (S^3^FEL). The reasons for increasing interest in the photocathode as the electron source are as following:

High quantum efficiency (QE) [9]. The quantum yield of the photocathode material and the available laser power should be high enough to generate the needed bunch charge required by the beam dynamics [10,11].

Long dark/charge lifetime [12]. The charge lifetime, determined by the photocathode and drive laser together, should be large enough at an acceptable length of time without interrupting the machine operation to change or rejuvenate the photocathode [13,14].

Low intrinsic emittance [15]. With the development of photocathode injectors, the intrinsic emittance of photocathode has become the main part of the final electron beam emittance and directly sets the minimum achievable emittance from the start. For an ultra-low emittance beam required application such as XFEL, it is critical to use a low intrinsic emittance photocathode or eliminate the intrinsic emittance of the photocathode with different methods [16,17,18,19,20,21,22].

Among the different kinds of photocathodes, the metallic photocathode is robust and has low intrinsic emittance, but relatively low QE. On the other hand, the metallic photocathode must be illuminated by a UV laser that cannot deliver the bunch charge for a high repetition rate/CW XFEL [23]. Therefore, a semiconductor photocathode is the only choice for a high repetition rate/CW XFEL. The first choice is Cs_2_Te as it has a high QE and long lifetime [24,25,26]. The bialkali photocathode is chosen as the backup for its high QE and low intrinsic emittance [27].

In this contribution, the recent development of a semiconductor photocathode in China is introduced, including the deposition systems, the activation recipe, and the characterizations of the photocathodes. Besides, the cathode performance in guns and the study of emission physics are also included.

## 2. Materials and Methods

Various semiconductor photocathode fabrication systems mainly for Cs_2_Te [26,28,29,30,31,32], bialkali (K_2_CsSb, K_2_NaSb) [33,34,35,36,37] and GaAs [14,38,39,40], have been built in different institutes in China and the growth procedures and characterizations of semiconductor photocathodes have been investigated.

### 2.1. Cs_2_Te Photocathode

The Cs_2_Te deposition systems are designed by local institutes in China and built/commissioned by civil vendors. The vacuum of these systems can reach as low as 10^−9^ Pa level by combining locally built chambers with imported vacuum devices such as a crystal monitor, UHV manipulator, NEG pump, vacuum gauge and controller, UHV valve, residual gas analyzer (RGA), etc. During past years, the vendors had made great progress in the UHV chamber, including chamber fabrication and post-processing. However, the design of chambers still needs to be upgraded.

**Figure 1:**
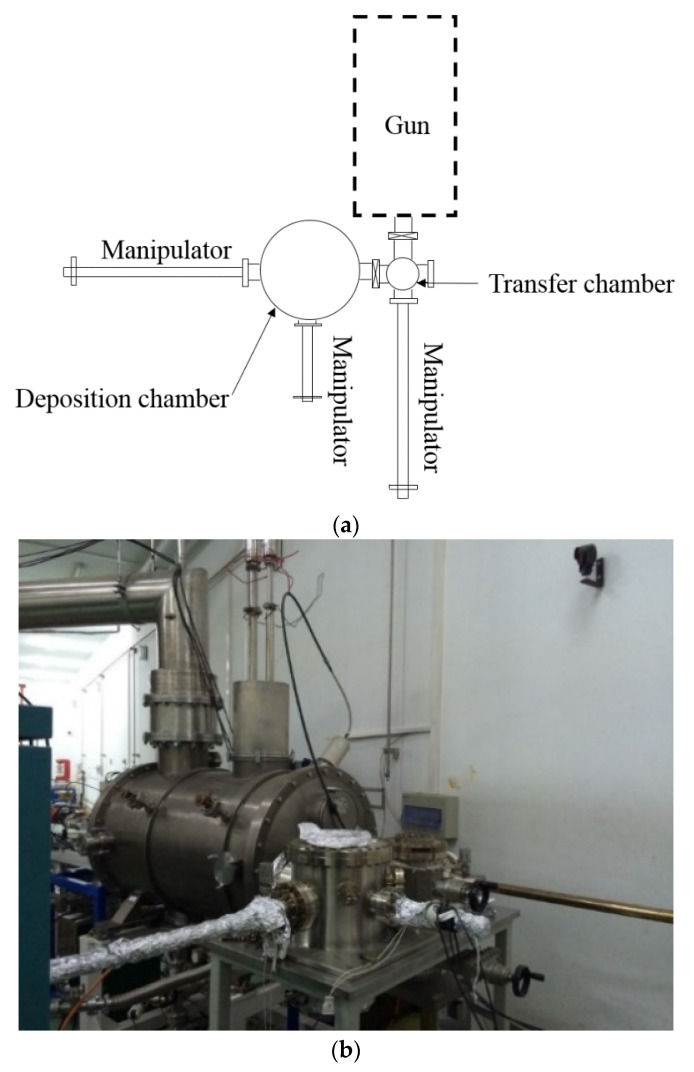
The schematic (**a**) and picture (**b**) of the Cs_2_Te deposition system at PKU.

The first fabricated system for Cs_2_Te photocathode in China was built in 2002 at Peking University (PKU) as shown in Figure 1 [41]. Since then, high QE Cs_2_Te photocathodes have been fabricated. The improved recipe for Cs_2_Te photocathode growth is as follows [42]:

(1) The substrate is heated at about 300 °C for more than 4 h to release the adsorbed gas.
(2) About 4~6 nm Te thin film is deposited on the substrate at 120 °C.
(3) The Cs activation starts when Te deposition is finished. The photocurrent is monitored during the cesiation process and the photocathode is illuminated by UV light. When the QE reaches plateau, the Cs source is turned off and the cathode will slowly cool down to room temperature. The QE of the fresh Cs_2_Te photocathode grown on Mo and stainless steel is about 10 % (@266 nm UV laser).

SHINE and S^3^ FEL have chosen very high frequency (VHF) NCRF gun [43,44] and Cs_2_Te photocathode as the baseline [45,46,47]. Therefore, Shanghai Advanced Research Institute (SARI, host of SHINE project), Dalian Institute of Chemical Physics (DICP), and Tsinghua University (THU) have built their Cs_2_Te deposition systems and started the recipe research separately. The QE of Cs_2_Te photocathodes fabricated at SARI and THU have achieved around 6% (@266 nm) and no QE measurement has been reported at DICP. Up to now, there are no beam test results published by more than three institutes.

**Figure 2:**
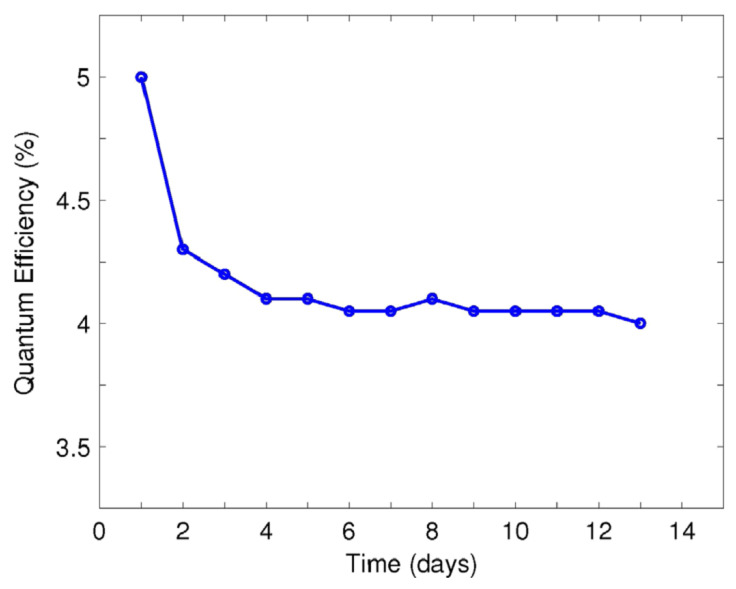
The QE of the Cs_2_Te photocathode in the PKU DC-SRF-I photoinjector [48]. Reproduced with the permission from reference [48].

No other gun test results are published for Cs_2_Te photocathode except PKU [48]. The Cs_2_Te photocathode performed well in the DC-SRF-I photoinjector during the beam experiment [41,49]. During the beam experiments of the DC-SRF-I photoinjector [48,50,51,52], the QE of Cs_2_Te photocathode was stabilized at 4% (@266 nm) in the photoinjector for months as shown in Figure 2. The electric field on the surface of the cathode is about 5 MV/m and the peak electric field is less than 13 MV/m. During the beam experiment, the E_acc_ of the SRF cavity in the DC-SRF-I photoinjector reached 14.5 MV/m in CW mode and 17 MV/m in pulse mode. The loss of Cesium during the transport of the cathode from deposition chamber to the gun is essential for keeping a long lifetime of the cathode. Therefore, the Cs_2_Te photocathode is reactivated before being transferred. The bunch charge was about 6~13 pC and the repetition rate was 81.25 MHz. The injector worked in pulse mode and the pulse repetition rate was 10 Hz. The average beam current was 1 mA in the macropulses with a length of 7 ms (duty factor 7%) and can be kept at about 0.5 mA for routine operation. The normalized transverse emittance of the delivered beam is 3.0 mm.mrad [48]. No dark current was observed by measuring with a faraday cup at the exit of the injector when keeping the DC high voltage and RF field on and without drive laser. The dark current was lower than 1 nA considering the measurement limitation.

### 2.2. Bialkali (K_2_CsSb, K_2_NaSb) Photocathode

The first K_2_CsSb photocathode system [53,54] in China was designed by the Shanghai Institute of Applied Physics (SINAP, now SARI) in 2012, where the first K_2_CsSb photocathode was grown with this system. The vacuum is as low as 10^−9^ Pa, which is similar to that of bialkali deposition systems at other laboratories around the world [10,12,18,36,55,56].

The deposition recipe of K_2_CsSb photocathode has been investigated at SARI since 2014. The recipe is as follows [57]:

(1) The Mo substrates is degassed at 370 °C for six hours to release the absorbed gas such as H_2_, O_2_, N_2_ and H_2_O. The metal oxide and old photocathode thin film can also be removed during the heating process.
(2) The substrate temperature is stabilized at about 75 °C, and Sb is evaporated to about 10 nm. The deposition rate is about 0.01 nm/s.
(3) Co-deposition method is adopted at SARI. The temperature of the substrate is kept at 75 °C, and the K and Cs is evaporated together at a ratio of about 1:1. The heating power of the sources should be controlled in time. The K and Cs sources are turned off when the photocurrent reached a plateau. After preparation, the photocathode is cooled down to room temperature.

**Figure 3:**
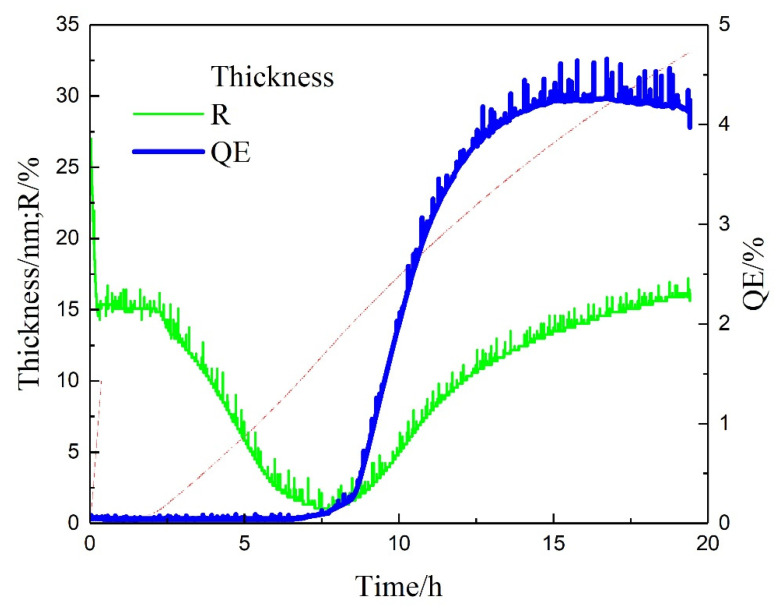
The activation process of K_2_CsSb photocathode at SARI. “R” is the reflectivity of the thin film during deposition, and the drive laser wavelength is 532 nm [57].

As shown in Figure 3, the activation process cost about 10 h. The reason for such a long activation process may be caused by the long distance (80 mm) between sources and substrate. During the activation process, the reflectivity of the thin film is monitored constantly. The reflectivity decreases with the increasing thickness of Sb. With the deposition of K and Cs, the reflectivity increased at the beginning and then decreases. When the reflectivity decreases to the lowest, the photocurrent starts to increase. After the QE reaches the plateau, the reflectivity stabilizes too. If K and Cs continue to deposit and the reflectivity will decrease, the QE will decrease too, which means that the reaction stopped and no new photocathode is formed. The reflectivity is a useful method to monitor the growth process of the bialkali photocathode.

**Figure 4:**
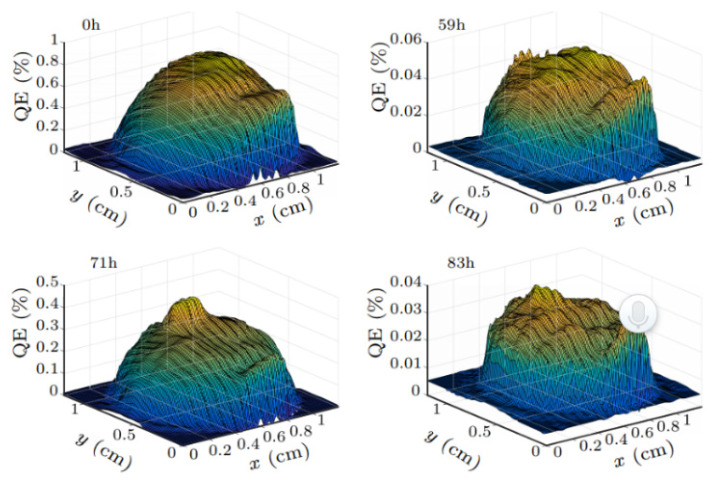
The QE (@532 nm) map of photocathode before and after thermal annealing at SARI [57].

The temperature dependence of QE of the K_2_CsSb photocathode during the activation process has been studied by SARI researchers [57]. The decayed photocathode could be recovered by heating without Cs atmosphere. Heating at 88 °C for 2 h can recover 50% of its original QE. But the photocathode QE after thermal annealing decreases faster. During the measurement of the bialkali photocathode lifetime, the QE at different time annealing are measured. Figure 4 shows the photocathode QE map before and after thermal annealing. The QE decreased and the uniformity became worse after thermal annealing, which means that some elements were missed during the deposition process.

**Figure 5:**
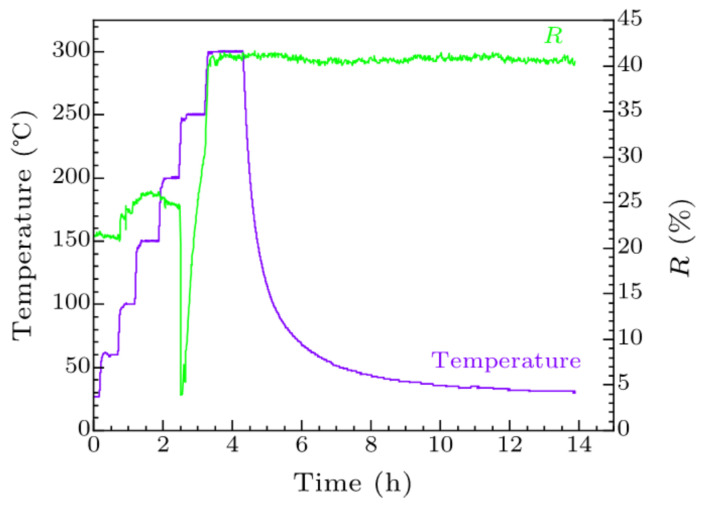
The substrate reflectivity and temperature during the heating removal process of K-Cs-Sb photocathode [57].

By heating the substrate at 300 °C [57], the low QE K-Cs-Sb photocathode can be removed from the substrate. The reflectivity of the degraded photocathode is monitored during the removal process. During the heating process (as shown in Figure 5), the reflectivity increases first and then decrease, finally increase to a stable value. The different reflectivity values corresponds to different compounds in the substrate and will be analyzed using in-situ X-ray in the future. When the reflectivity does not change any more, the used photocathode layer is completely removed. This is a useful method to reuse the substrate plug without breaking the vacuum. The used substrates have to be mechanically polished at many laboratories around the world, which cost a lot of time and the surface roughness may be influenced during the polishing process. Complete removal can be characterized with the reflectivity method. According to the results at SARI, no big difference on QE is observed from the heating removal and mechanically polished substrates.

**Figure 6:**
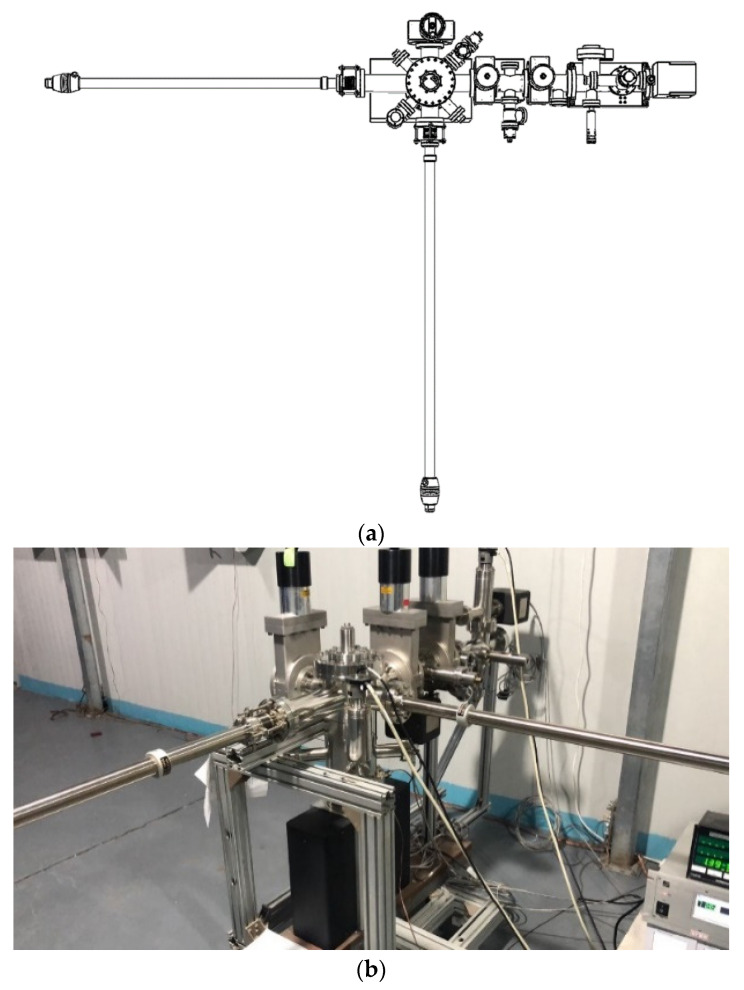
The schematic (**a**) and picture (**b**) of the transferring chamber, load lock and the transport suitcase at SARI.

INFN (National Institute for Nuclear Physics, Italy) type substrates and suitcases are used at SARI [28,58]. Figure 6 shows the suitcase, load lock and transferring chamber, which will be used in the SHINE project at SARI. The suitcase comprised of a storage chamber, a 20 L/s sputtering ion pump and a 400 L/s SAES NEG pump, an anode to measure the photocurrent from a viewport in one side of the storage chamber. Up to four plugs can be stored in the photocathode carrier in the suitcase. After the photocathodes is fabricated, the suitcase is demounted from the deposition chamber and connected to the transfer chamber through the load lock. The load lock comprised of an ion pump and an all-metal angle valve. The load lock is heated up to 200 °C to derive UHV before the two gate valves can be opened. Two UHV manipulators is mounted on the transfer chamber. One manipulator is to transfer the photocathode carrier between the suitcase and transfer chamber. The other manipulator is to transfer the photocathode in and out of the gun. The vacuum level is kept at low 10^−9^ Pa level in these chambers.

The Institute of High Energy Physics [59] (IHEP), China Academy of Engineering Physics (CAEP), and PKU started to design and build their K_2_CsSb photocathode systems from 2014. THU, DICP are also planning to build their deposition systems for bialkali (K_2_CsSb or K_2_NaSb) photocathode.

**Figure 7:**
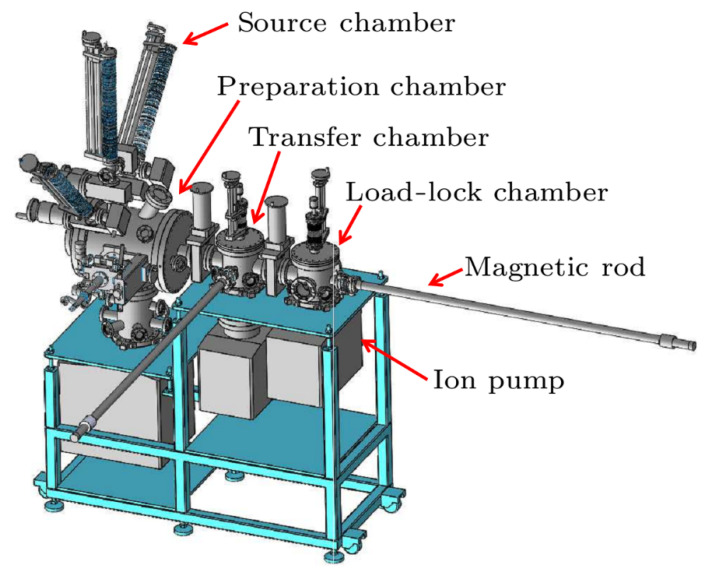
The bialkali photocathode deposition system at IHEP [59].

The deposition chamber and the recipe for K_2_CsSb photocathode at IHEP (as shown in Figure 7) and CAEP are similar to that of SARI. During preliminary experiment, the QE of K_2_CsSb photocathode was about 1%. The deposition chambers at CAEP are mainly used for Cs_2_Te preparation at present after a test of K_2_CsSb photocathode fabrication.

**Figure 8:**
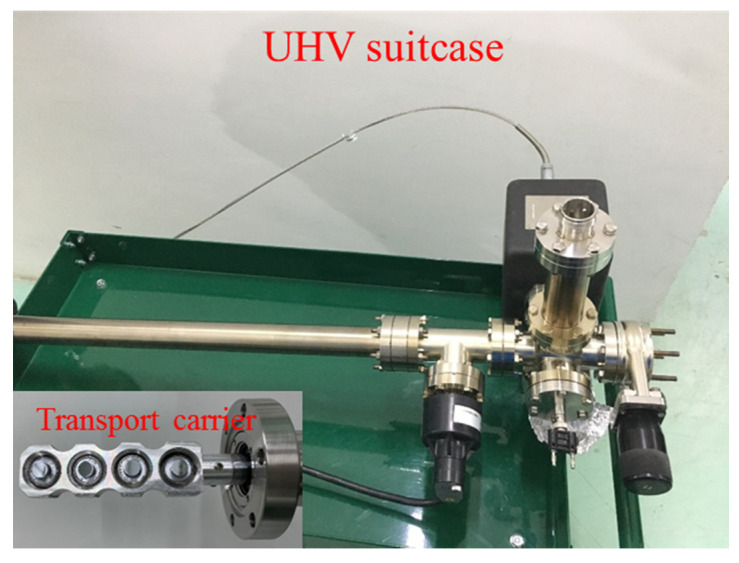
The UHV suitcase and transport carrier at PKU.

At PKU, a deposition chamber with four arms has been designed and manufactured since 2017. A different recipe with very fast cesiation process was adopted and optimized. The advantage of the verified recipe is that the K_2_CsSb photocathodes can have a longer lifetime. Recent experiments show that the QE of K_2_CsSb photocathodes are 4.1–7.4% and no degradation has been observed in DC-SRF gun for 2 weeks. The photocathodes were transported from deposition chamber to DC-SRF gun by a suitcase at a vacuum level of 9 × 10^−9^ Pa as shown in Figure 8.

### 2.3. GaAs Photocathode

There are two GaAs photocathode deposition systems at IHEP and CAEP in China. The vacuum of these two systems is about 2~4 × 10^−10^ Pa. Sputtering ion pumps (200~400 L/s, homemade) and NEG pumps (2000~4000 L/s, SAES) are used in these systems with Leybold IE514 as the vacuum monitor.

The fabrication of a GaAs photocathode has been accomplished by IHEP and CAEP. 10% QE (@532 nm) and weeks of lifetime have been derived during past years, which is reliable and repeatable. At present, no polarized electron source based on superlattice GaAs has been investigated for an accelerator-based project in China [60,61,62]. The typical recipe for high QE GaAs photocathode at CAEP is as follows:

(1) The GaAs substrate is cleaned by high temperature heating [63] to remove the residual oxygen and other impurity on the surface of the GaAs sample. The time for the high temperature cleaning is about 45 min.
(2) The Cs source is heated to release Cs atom to activate the GaAs. The photocurrent will increase after 20 min. The vacuum of the deposition chamber will decrease to 1 × 10^−7^ Pa from 4 × 10^−8^ Pa.
(3) When the photocurrent reaches the plateau, the Cs source is shut off and oxygen is brought into the chamber to increase the photocurrent. When the photocurrent decreased, shut off oxygen and open the Cs source again. Then repeated the above process for several cycles to the highest photocurrent.

**Figure 9:**
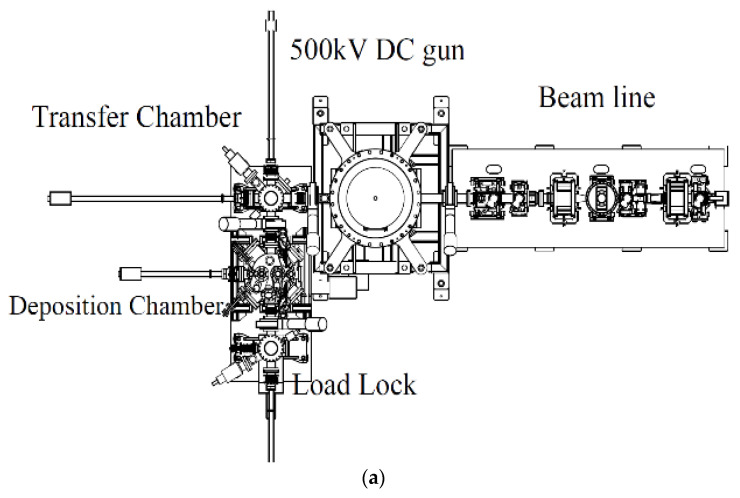

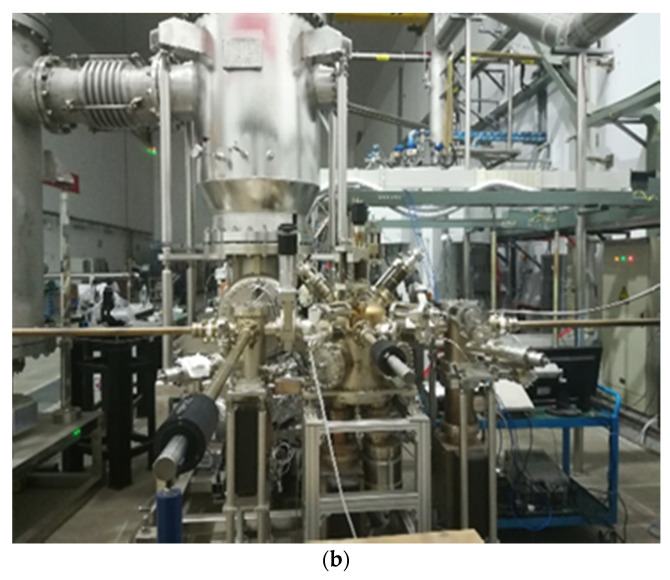
The schematic (**a**) and picture (**b**) of the GaAs photocathode deposition system at CAEP.

During the heating removal process, the temperature distribution on the GaAs photocathode is derived with the ANSYS program. The QE distribution of the GaAs photocathode fabricated is relevant with the temperature distribution in the reference [63]. The HV DC gun (500 kV) is the key element of the THz-FEL facility at CAEP. With the QE of about 5% in DC gun, an average CW beam current of 5 mA was accelerated to 8 MeV in the SRF cryomodule [64]. The normalized emittance was about 6 mm.mrad at the CW 4.8 mA average current. The intrinsic emittance of GaAs photocathode was measured to be 0.6 mm.mrad/mm from the beam line at CAEP as shown in Figure 9a,b. The factors that affect the GaAs photocathode operation lifetime in the high voltage DC gun are investigated. The vacuum and ion backbombardment are the principle factors affecting the lifetime. The effects and limitations of the beam off-axis emission to suppress the ion back bombardment is simulated at CAEP [65]. The simulation suggested that the ions mainly bombard the electrostatic center of the photocathode and 95% can be reduced if the electron is excited off-axis. The QE maps are measured before and after the DC gun (shown in Figure 9b) CW operation in both the on and off-axis emission, which agrees well with the simulation results.

At IHEP, a GaAs photocathode was used as the electron source in the 500 kV DC gun. During the beam experiment, the QE of GaAs photocathode was about 5% (@532 nm) and a 5 mA average current CW beam was derived. Now the DC gun at IHEP is used as the high energy photon source (HEPS) test bench, which is used to test the SRF cavities and cryo-modules for XFEL and the proposing circular electron-positron collider (CEPC).

## 3. Physics of the Emission Process

The researchers at PKU [55], SARI [53], and THU [66] have simulated the emission process of the alkali antimony photocathodes.

**Figure 10:**
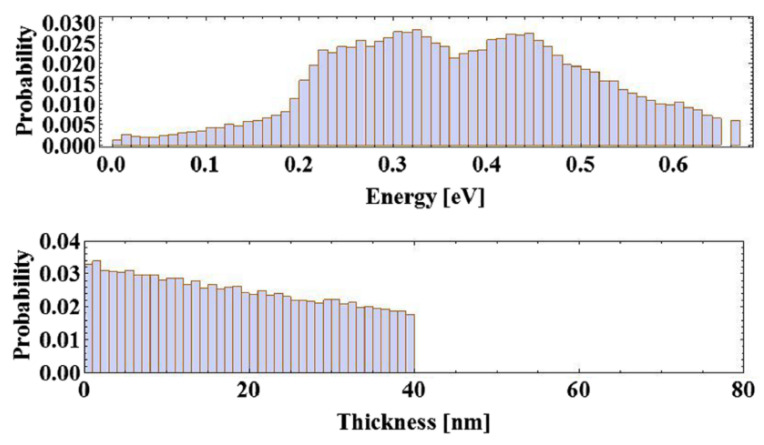
(**top**) The histogram of the electron-energy distribution in the conduction band excited by 2.32 eV laser photons at room temperature. The Y-axis is the probability distribution of electrons at different energies. (**bottom**) The longitudinal distribution of excited electrons along the cathode’s thickness (40 nm) with exponential decay [55].

**Figure 11:**
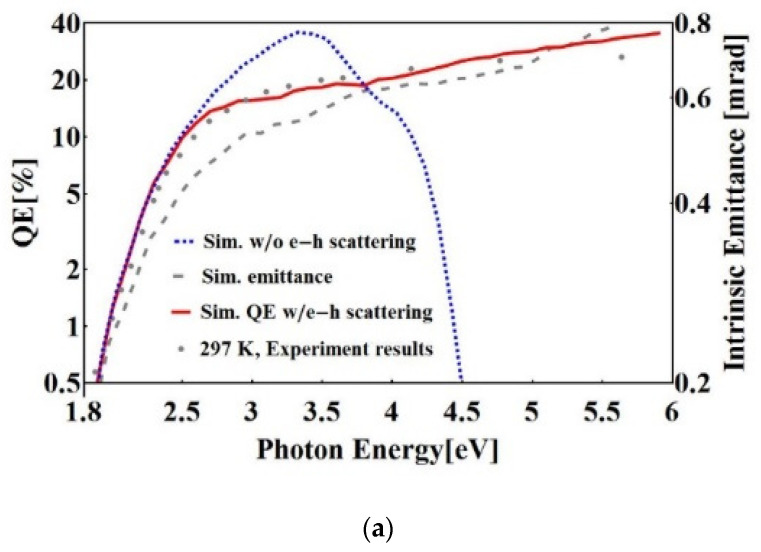

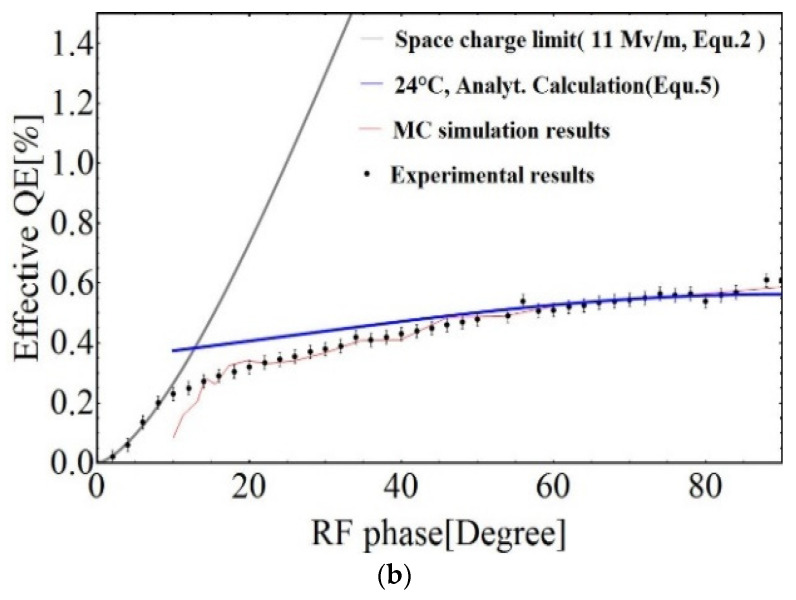
(**a**) The simulation results of spectral response with and without electron–hole (e–h) scattering, and intrinsic emittance at room temperature. The dots are the experimental results of the spectral response of the K_2_CsSb photocathode at room temperature [55]. (**b**) The Schottky effect on the cryo-cooled K_2_CsSb photocathode in the 704 MHz SRF gun at Brookhaven National Lab (BNL) [68]. Reproduced with the permission from reference [68].

The Monte Carlo simulation [67] is based on the Spicer’s three step model. The initial electron distribution is derived with the convolution of the density of states (DoS) in the valence band and in the conduction band. The initial electron distribution is calculated with the localized spherical-wave method. In Figure 10, the percentage of electrons with different energy and the energy distribution in the thickness direction is calculated. The number of the photons/electrons used in the simulation is 100,000. The spectral response, intrinsic emittance, response time, and cryogenic performance can be derived from this code as shown in Figure 11a, which agrees well the experimental results [55]. The spectral response is simulated with and without electron–hole scattering, respectively, which will greatly influence the results by changing the energy loss during the electron transport step in the conduction band, especially in the high photon energy range [6]. The difference is that in the high energy part, the QE will decrease if the electron-hole scattering is included. The emission time of the electrons, which is an important factor for the bunch length, is less than 200 fs from the simulation, which agree well with the experimental results.

The Schottky effect [68] on cryo-cooled K_2_CsSb photocathode at high gradient in the SRF gun is also simulated, which has a nice correlation with the experimental data as shown in Figure 11b [68]. When the RF phase changes, the electric field applied on the surface of the cryo-cooled K_2_CsSb photocathode will change, and the surface barrier will be lower according to the Schottky effect. Therefore, the effective QE will grow with the RF phase. At the beginning of the RF phase scan, the photocurrent is limited by the space charge limit. The author explained this phenomenon with an analytical model and the Monte Carlo simulation [68], which agreed well with the experimental results. This is helpful to understand the mechanism of the cryo-effect on the intrinsic emittance reduction of the K_2_CsSb photocathode and is essential to derive an ultralow intrinsic emittance photocathode. The heterojunction photocathodes (K_2_NaSb/Cs_3_Sb, GaAs/Cs_2_Te, and GaAs/Cs_3_Sb) are also simulated at PKU with this code [69]. These heterojunction photocathodes aim to find the physics of the emitted electrons and to achieve low emittance, high QE, and a long lifetime photocathode, especially for a long lifetime polarized photocathode [70].

**Figure 12:**
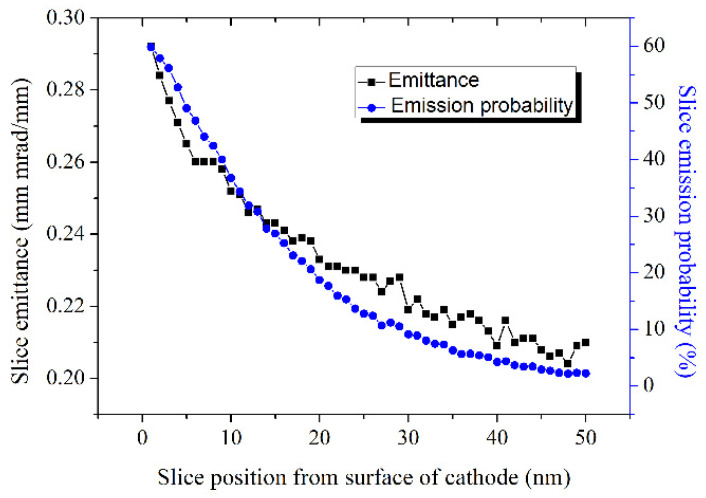
The black line shows the simulated intrinsic emittance of K_2_CsSb photocathode with the assumption that all electrons are emitted from one slice (thickness is 1 nm). The blue line indicates the emission probability [47].

At SARI [53], the researcher gave a special view on the emission process of K_2_CsSb photocathode as shown in Figure 12: the electron bunch is divided by “electron slice” inside the cathode material, which separately contributes to the intrinsic emittance, just as the slice emittance contributes to the final emittance [71]. The scattering probability increases with the transport distance and more electron energy would lose during the scattering process. The emission probability and transverse momenta of the electrons would decrease with the photocathode depth. Therefore, the distribution of the photoelectrons to final QE and intrinsic emittance decreases with increasing depth. This is very helpful to understand the mechanism of the intrinsic emittance, which in turn can help to derive a high brightness electron beam by adjusting the initial distribution of the photoelectron along the depth direction.

**Figure 13:**
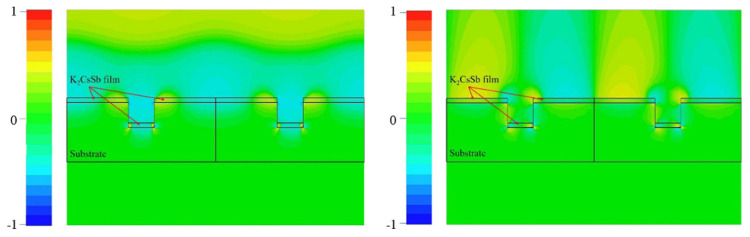
Electric field distribution of PNF over cross section. Left and right images show electric field distributions of X and Y components of PNF, respectively [73].

To enhance the electron emission, the surface polarized plasmons (SPPs) are also investigated at SARI [72,73,74,75,76]. The silver substrate with a nanopattern is employed to achieve wave vector matching and the SPPs is excited between the substrate and photocathode [73]. The nanopattern on the silver substrate (the width, depth, and the thickness of the photocathode) is optimized by CST studio to achieve the needed SPPs excited by specific wavelength. When the absorption rate of the incident laser to photocathode with nanopattern rises to 95%, the SPPs are successfully excited. The electromagnetic field near the surface between the substrate and the photocathode is redistributed, periodic and aligned with the nanopattern structure. Figure 13 shows the PNF of two unit cells with a phase of 90 degree caused by the SPPs. The electromagnetic field is enhanced by the SPPs in the interface between the substrate and the photocathode film. Hence the increased absorption and the designed PNF can help to improve the photocathode’s performance, such the QE and intrinsic emittance.

**Figure 14:**
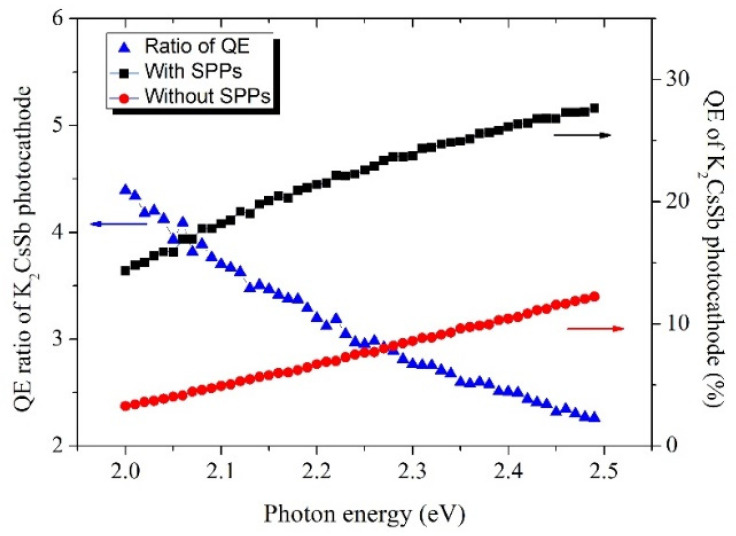
Simulated data showing the relationship between QE of K_2_CsSb photocathode and incident photon energy with/without surface polarized plasmons (SPPs). Black and red curves are QEs of K_2_CsSb photocathodes with and without SPPs, respectively; the blue curve represents the ratio of QE of K_2_CsSb photocathodes with SPPs to that of K_2_CsSb photocathodes without SPPs [73].

The results of the Monte Carlo simulations in Figure 14 showed that with SPPs, the absorption efficiency for 532 nm laser would rise two times as before. This will help to raise the QE of the K_2_CsSb photocathode, and the emittance can be kept at 0.5~0.8 mm.mrad/mm. These results show that part of the emittance growth is caused by plasmonic near field (PNF). Therefore, this can be suppressed and new possibilities for tailoring the nature of materials with improved performance can be achieved from the simulation results. The simulated results show that the QE of a K_2_CsSb photocathode with SPPs is 2–3 times that of the photocathode without SPPs [73]. Furthermore, the intrinsic emittance of the photocathode remains substantially constant, which means it is possible to obtain a photocathode with high QE and low emittance at the same time.

**Figure 15:**
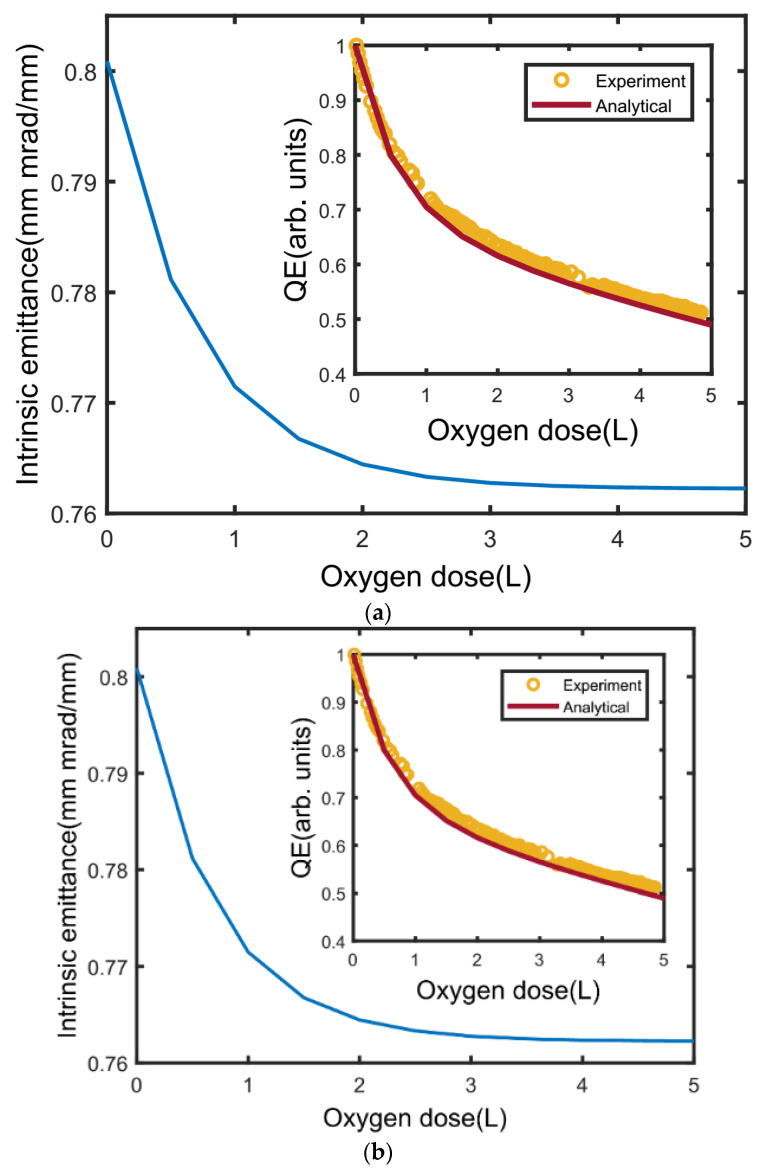
(**a**) Experimental and analytical model of QE’s decay along with oxygen dose in the chamber; (**b**) QE and intrinsic emittance evolution of Cs_3_Sb photocathode with incident photon energy at 1.8 and 1.9 eV, respectively [66].

The cryogenic performance of the Cs_3_Sb cathode and its degradation mechanism [66], caused by residual gas in the chamber, is also simulated at THU. The acceptor level of the Cs_3_Sb photocathode is considered in the Monte Carlo model. The change of the acceptor level is considered the reason for the decay of the Cs_3_Sb cathode at a cryogenic temperature. The model agrees well with the experimental results achieved by Cornell [17]. The impact of residual gas on intrinsic emittance is also investigated by the dynamic model [77]. The main mechanism in the dynamical model is that the electron affinity and electron transmission probability will change with the electron affinity affected by the Cs atoms reacting with residual gas on the surface of the cathode. The simulation results are shown Figure 15a. The analytical results agree well with the experiment of QE degradation in reference, where the QE degrades to about 50% of its original value. The cathode intrinsic emittance decreases a little and stabilizes at about 0.762 mm.mrad/mm. The author also discussed when the incident photon energy is lower than the threshold under typical DC gun and RF gun conditions (4 and 50 MV/m, respectively) [78,79]. The QE degrades in both cases while the intrinsic emittance differs a lot, and the main reason is the different contribution to the acceptor level under these two electric fields. At 4 MV/m, the photoelectrons mainly come from the acceptor level and at 50 MV/m from the valence band. In addition, the simulated results suggest that the intrinsic emittance of the near-threshold emission (driven by a 690 nm laser) of the Cs_3_Sb photocathode is better than UV laser-drived metal cathode when the QE remains above an acceptable minimum level. This is helpful in high brightness electron sources which low intrinsic emittance and high QE are needed at the same time.

## 4. Conclusions

In China, research on semiconductor photocathodes has been attracting growing interest. Different kinds of deposition systems with imported UHV devices for a semiconductor photocathode such as Cs_2_Te, bialkali and GaAs have been built. The vacuum level of deposition systems is in the 10^−8^~10^−10^ Pa level. The fabrication recipe, transport, gun test and emission physics have been widely investigated by local institutes and universities. The reliability and consistency of deposition systems need to be improved in the future.

## 5. Forward Look

In the following years, high QE and long lifetime semiconductor photocathodes will be used for scientific projects by institutes in China, especially the Cs_2_Te photocathode for the XFEL application. Research on reducing the intrinsic emittance will be studied, such as SPPs, cryogenic photocathodes, heterojunction photocathodes, etc. The semiconductor photocathode will be tested in different type of guns and injectors (DC, RF, and SRF) to derive the required electron beam. The emission physics will also be studied to understand and improve the performance of the semiconductor photocathode.

## References

[1] Xin T., Brutus J.C., Belomestnykh S.A., Ben-Zvi I., Boulware C.H., Grimm T.L., Hayes T., Litvinenko V.N., Mernick K., Zaltsman A. (2016). Design of High-bunch-charge 112 MHz Superconducting RF photoemission electron source. Rev. Sci. Instrum..

[2] Brinkmann R., Schneidmiller E.A., Yurkov M.V. (2010). Possible operation of the European XFEL with ultra-low emittance beams. Nucl. Instrum. Methods Phys. Res. Sect. A Accel. Spectrom. Detect. Assoc. Equip..

[3] Zhao Z. XFEL Projects in China. Proceedings of the 29th Linear Accelerator Conference.

[4] Chen X. An electron ion collider in China. Proceedings of the 9th APCTP-BLTP JINR Joint Workshop at Kazakhstan.

[5] Gulliford C., Bartnik A., Bazarov I. (2016). Multiobjective optimizations of a novel cryocooled dc gun based ultrafast electron diffraction beam line. Phys. Rev. Accel. Beams.

[6] Xiang D., Du Y.C., Yan L.X., Li R.K., Huang W.H., Tang C.X., Lin Y.Z. (2009). Transverse phase space tomography using a solenoid applied to a thermal emittance measurement. Phys. Rev. Spec. Top.-Accel. Beams.

[7] Xiang D., Fu F., Zhang J., Huang X., Wang L., Wang X., Wan W. (2014). Accelerator-based single-shot ultrafast transmission electron microscope with picosecond temporal resolution and nanometer spatial resolution. Nucl. Instrum. Meth. A.

[8] Petrushina I., Litvinenko V.N., Jing Y., Ma J., Pinayev I., Shih K., Wang G., Wu Y.H., Altinbas Z., Brutus J.C. (2020). High-brightness continuous-wave electron beams from superconducting radio-frequency photoemission gun. Phys. Rev. Lett..

[9] Dowell D.H. Photocathode properties and requirements for photoinjectors. Proceedings of the EuroFEL Workshop.

[10] Schmeißer M.A.H., Mistry S., Kirschner H., Schubert S., Jankowiak A., Kamps T., Kühn J. (2018). Towards the operation of Cs-K-Sb photocathodes in superconducting rf photoinjectors. Phys. Rev. Accel. Beams.

[11] Wang E., Rao T., Ben-zvi I. (2014). Enhancement of photoemission from and postprocessing ofK2CsSbphotocathode using excimer laser. Phys. Rev. Spec. Top.-Accel. Beams.

[12] Wang E., Litvinenko V.N., Pinayev I., Gaowei M., Skaritka J., Belomestnykh S., Ben-Zvi I., Brutus J.C., Jing Y., Biswas J. (2021). Long lifetime of bialkali photocathodes operating in high gradient superconducting radio frequency gun. Sci. Rep..

[13] Mammei R.R., Suleiman R., Feingold J., Adderley P.A., Clark J., Covert S., Grames J., Hansknecht J., Machie D., Poelker M. (2013). Charge lifetime measurements at high average current using aK_2_CsSbphotocathode inside a dc high voltage photogun. Phys. Rev. Spec. Top.-Accel. Beams.

[14] Rahman O., Wang E., Ben-Zvi I., Biswas J., Skaritka J. (2019). Increasing charge lifetime in dc polarized electron guns by offsetting the anode. Phys. Rev. Accel. Beams.

[15] Prat E., Bettoni S., Braun H.-H., Ganter R., Schietinger T. (2015). Measurements of copper and cesium telluride cathodes in a radio-frequency photoinjector. Phys. Rev. Spec. Top.-Accel. Beams.

[16] Cultrera L., Gulliford C., Bartnik A., Lee H., Bazarov I. (2017). Rb based alkali antimonide high quantum efficiency photocathodes for bright electron beam sources and photon detection applications. J. Appl. Phys..

[17] Lee H., Cultrera L., Bazarov I. (2016). Intrinsic emittance reduction in transmission mode photocathodes. Appl. Phys. Lett..

[18] Cultrera L., Gulliford C., Bartnik A., Lee H., Bazarov I. (2016). Ultra low emittance electron beams from multi-alkali antimonide photocathode operated with infrared light. Appl. Phys. Lett..

[19] Maxson J., Lee H., Bartnik A.C., Kiefer J., Bazarov I. (2015). Adaptive electron beam shaping using a photoemission gun and spatial light modulator. Phys. Rev. Spéc. Top.-Accel. Beams.

[20] Maxson J., Cultrera L., Gulliford C., Bazarov I. (2015). Measurement of the tradeoff between intrinsic emittance and quantum efficiency from a NaKSb photocathode near threshold. Appl. Phys. Lett..

[21] Karkare S., Boulet L., Cultrera L., Dunham B., Liu X., Schaff W., Bazarov I. (2014). Ultrabright and ultrafast III–V semiconductor photocathodes. Phys. Rev. Lett..

[22] Wang Y., Mamun M.A., Adderley P., Bullard B., Grames J., Hansknecht J., Hernandez-Garcia C., Kazimi R., Krafft G.A., Palacios-Serrano G. (2020). Thermal emittance and lifetime of alkali-antimonide photocathodes grown on GaAs and molybdenum substrates evaluated in a −300 kV dc photogun. Phys. Rev. Accel. Beams.

[23] Dowell D.H., Schmerge J.F. (2009). Quantum efficiency and thermal emittance of metal photocathodes. Phys. Rev. Spéc. Top.-Accel. Beams.

[24] Xiang R., Arnold A., Buettig H., Janssen D., Justus M., Lehnert U., Michel P., Murcek P., Schamlott A., Schneider C. (2010). Cs2Te normal conducting photocathodes in the superconducting rf gun. Phys. Rev. Spéc. Top.-Accel. Beams.

[25] Rimjaem S., Stéphan F., Krasilnikov M., Ackermann W., Asova G., Bahr J., Gjonaj E., Grabosch H., Hakobyan L., Hanel M. (2011). Optimizations of transverse projected emittance at the photo-injector test facility at DESY, location Zeuthen. Nucl. Instrum. Meth. A.

[26] Terunuma N., Murata A., Fukuda M., Hirano K., Kamiya Y., Kii T., Kuriki M., Kuroda R., Ohgaki H., Sakaue K. (2009). Improvement of an S-band RF gun with a Cs2Te photocathode for the KEK-ATF. Nucl. Instrum. Meth. A.

[27] Vecchione T., Ben-Zvi I., Dowell D.H., Feng J., Rao T., Smedley J., Wan W., Padmore H.A. (2011). A low emittance and high efficiency visible light photocathode for high brightness accelerator-based X-ray light sources. Appl. Phys. Lett..

[28] Filippetto D., Qian H., Sannibale F. (2015). Cesium telluride cathodes for the next generation of high-average current high-brightness photoinjectors. Appl. Phys. Lett..

[29] Wisniewski E.E., Velázquez D., Yusof Z., Spentzouris L., Terry J., Sarkar T.J., Harkay K. (2013). Kelvin probe studies of cesium telluride photocathode for AWA photoinjector. Nucl. Instrum. Methods Phys. Res. Sect. A Accel. Spectrom. Detect. Assoc. Equip..

[30] Sakaue K., Hayano H., Kashiwagi S., Kuroda R., Masuda A., Suzuki T., Takatomi T., Terunuma N., Urakawa J., Washio M. (2011). Cs–Te photocathode RF electron gun for applied research at the Waseda University. Nucl. Instrum. Meth. B.

[31] Kuroda R., Ogawa H., Sei N., Toyokawa H., Yagi-Watanabe K., Yasumoto M., Koike M., Yamada K., Yanagida T., Nakajyo T. (2008). Development of Cs2Te photocathode rf gun system for compact THz SASE-FEL. Nucl. Instrum. Meth. A..

[32] Gaowei M., Sinsheimer J., Strom D., Xie J., Cen J., Walsh J., Muller E., Smedley J. (2019). Codeposition of ultrasmooth and high quantum efficiency cesium telluride photocathodes. Phys. Rev. Accel. Beams.

[33] Kimoto T., Arai Y., Nagayama K. (2017). Experiments for improving fabrication, recovery and surface-protection of Cs3Sb photocathode. Appl. Surf. Sci..

[34] Schubert S., Wong J., Feng J., Karkare S., Padmore H., Oses M.R., Smedley J., Muller E., Ding Z., Gaowei M. (2016). Bi-alkali antimonide photocathode growth: An X-ray diffraction study. J. Appl. Phys..

[35] Mamun M.A., Hernández-García C., Poelker M., Elmustafa A.A. (2015). Correlation of CsK2Sb photocathode lifetime with antimony thickness. APL Mater..

[36] Schubert S., Oses M.R., Ben-Zvi I., Kamps T., Liang X., Muller E., Muller K.O., Padmore H., Rao T., Tong X. (2013). Bi-alkali antimonide photocathodes for high brightness accelerators. APL Mater..

[37] Dunham B.M., Barley J., Bartnik A., Bazarov I., Cultrera L., Dobbins J., Hoffstaetter G., Johnson B., Kaplan R., Karkare S. (2013). Record high-average current from a high-brightness photoinjector. Appl. Phys. Lett..

[38] Hao G., Zhang Y., Jin M., Feng C., Chen X., Chang B. (2015). The effect of surface cleaning on quantum efficiency in AlGaN photocathode. Appl. Surf. Sci..

[39] Suntrup D.J., Gupta G., Li H., Keller S., Mishra U.K. (2014). Measurement of the hot electron mean free path and the momentum relaxation rate in GaN. Appl. Phys. Lett..

[40] Grames J., Suleiman R., Adderley P.A., Clark J., Hansknecht J., Machie D., Poelker M., Stutzman M.L. (2011). Charge and fluence lifetime measurements of a dc high voltage GaAs photogun at high average current. Phys. Rev. Spéc. Top.-Accel. Beams.

[41] Xiang R., Ding Y.T., Zhao K., Lu X.Y., Quan S.W., Zhang B.C., Wang L.F., Huang S.L., Lin L., Chen J. (2004). Experimental investigations of DC-SC photoinjector at Peking University. Nucl. Instrum. Meth. A.

[42] Dai J., Quan S.-W., Chang C., Liu K.-X., Zhao K. (2012). Cs 2 Te photocathode fabrication system at Peking University. Chin. Phys. C.

[43] Wells R.P., Ghiorso W., Staples J., Huang T.M., Sannibale F., Kramasz T.D. (2016). Mechanical design and fabrication of the VHF-gun, the Berkeley normal-conducting continuous-wave high-brightness electron source. Rev. Sci. Instrum..

[44] Sannibale F., Baptiste K.M., Cork C.W., De Santis S., Dickinson M., Doolittle L., Doyle J., Feng J., Filippetto D., Harris G. Status, plans and recent results from the apex project at LBNL. Proceedings of the IPAC 2014.

[45] Zhou F., Brachmann A., Emma P., Gilevich S., Huang Z. (2012). Impact of the spatial laser distribution on photocathode gun operation. Phys. Rev. Spéc. Top.-Accel. Beams.

[46] (2014). LCLS-II Final Design Report.

[47] Zhou F., Brachmann A., Decker F.J., Emma P., Gilevich S., Iverson R., Stefan P., Turner J. (2012). High-brightness electron beam evolution following laser-based cleaning of a photocathode. Phys. Rev. Spec. Top.-Accel. Beams.

[48] Quan S., Hao J., Lin L., Zhu F., Wang F., Feng L., Huang S., Wang Z., Wen X., Fan P. (2015). Stable operation of the DC-SRF photoinjector. Nucl. Instrum. Methods Phys. Res. Sect. A Accel. Spectrom. Detect. Assoc. Equip..

[49] Quan S., Zhu F., Hao J., Lu X., Zhang B., Xu W., Zhao K. (2010). 3.5-cell large grain niobium superconducting cavity for a dc superconducting rf photoinjector. Phys. Rev. Spéc. Top.-Accel. Beams.

[50] Wang Z.-W., Huang S.-L., Lin L., Zhao G., Quan S.-W., Liu K.-X., Chen J.-E. (2016). Drive laser system for the DC-SRF photoinjector at Peking University. Chin. Phys. C.

[51] Feng L.W., Lin L., Huang S.L., Quan S.W., Jiang T., Zhu P.F., Hao J.K., Zhu F., Wang F., Fu F. (2015). Ultrafast electron diffraction with megahertz MeV electron pulses from a superconducting radio-frequency photoinjector. Appl. Phys. Lett..

[52] Wang W.-J., Huang S.-L., Quan S.-W., Lin L., Liu K.-X. (2012). An emittance measurement device for a space-charge dominated electron beam. Chin. Phys. C.

[53] Li X.-D., Gu Q., Zhang M., Zhao M.-H. (2012). The QE numerical simulation of PEA semiconductor photocathode. Chin. Phys. C.

[54] Ding Z., Karkare S., Feng J., Filippetto D., Johnson M., Virostek S., Sannibale F., Nasiatka J., Gaowei M., Sinsheimer J. (2017). Temperature-dependent quantum efficiency degradation of K-Cs-Sb bialkali antimonide photocathodes grown by a triple-element codeposition method. Phys. Rev. Accel. Beams.

[55] Xie H., Ben-Zvi I., Rao T., Xin T., Wang E. (2016). Experimental measurements and theoretical model of the cryogenic performance of bialkali photocathode and characterization with Monte Carlo simulation. Phys. Rev. Accel. Beams.

[56] Mamun A.A., Elmustafa A.A., Hernández-García C., Mammei R., Poelker M. (2016). Effect of Sb thickness on the performance of bialkali-antimonide photocathodes. J. Vac. Sci. Technol. A.

[57] Li X.-D., Jiang Z.-G., Gu Q., Zhao M.-H., Guo L. (2020). Preliminary systematic study of the temperature effect on the K–Cs–Sb photocathode performance based on the K and Cs Co-evaporation*. Chin. Phys. Lett..

[58] Bisero D., Di Bona A., Paradisi P., Valeri S. (2000). K2Te photocathode growth: A photoemission study. J. Appl. Phys..

[59] Zhang F., Li X.-P., Li X.-S. (2019). Development of preparation systems with K 2 CsSb photocathodes and study on the preparation process. Chin. Phys. Lett..

[60] Wang E.D., Kewisch J., Ben-Zvi I., Burrill A., Rao T., Wu Q.O., Jain A., Gupta R., Holmes D., Zhao K. (2011). Heat load of a GaAs photocathode in an SRF electron gun. Chin. Phys. C.

[61] Zou J., Ge X., Zhang Y., Deng W., Zhu Z., Wang W., Peng X., Chen Z., Chang B. (2016). Negative electron affinity GaAs wire-array photocathodes. Opt. Express.

[62] Jin X., Ohki S., Ishikawa T., Tackeuchi A., Honda Y. (2016). Analysis of quantum efficiency improvement in spin-polarized photocathode. J. Appl. Phys..

[63] Xiao D.X., Li K., Pan Q. (2013). High temperature thermal cleaning for GaAs photocathode. High Power Laser Part. Beams.

[64] Kui Z., Chenglong L., Dai W., Xing L., Jianxin W., Dexin X., Lijun S., Tianhui H., Xuming S., Sifen L. (2018). Performance of the 2 × 4-cell superconducting linac module for the THz-FEL facility. Nucl. Instrum. Methods Phys. Res. Sect. A Accel. Spectrom. Detect. Assoc. Equip..

[65] Yang R.J., Li K., Xiao D., Wang J.X., Liu Y., Wang H.B., Wu D., Yang X.F. (2015). Beam off-axis emission in direct current high voltage photocathode gun. High Power Laser Part. Beams.

[66] Huang P.-W., Qian H., Du Y., Huang W., Zhang Z., Tang C. (2019). Photoemission and degradation of semiconductor photocathode. Phys. Rev. Accel. Beams.

[67] Marini J., Bell L.D., Shahedipour-Sandvik F. (2018). Monte Carlo simulation of III-nitride photocathodes. J. Appl. Phys..

[68] Xie H.-M., Wang E.-D., Liu K.-X. (2018). Analytical model and simulation of the Schottky effect on a cryo-cooled bialkali photocathode. Nucl. Sci. Tech..

[69] Droubay T.C., Chambers S.A., Joly A.G., Hess W.P., Németh K., Harkay K.C., Spentzouris L. (2014). Metal-insulator photocathode heterojunction for directed electron emission. Phys. Rev. Lett..

[70] Bae J.K., Cultrera L., Digiacomo P., Bazarov I. (2018). Rugged spin-polarized electron sources based on negative electron affinity GaAs photocathode with robust Cs2Te coating. Appl. Phys. Lett..

[71] Akira I., Takuya N., Mitsuhiro Y. (2017). Cut disk structure type RF-deflector for slice emittance measurement for RF-gun at SuperKEKB. Spec. Issue Fifth Int. Symp. Innov. Nucl. Energy Syst..

[72] Jiang Z., Li X., Huang D., Zhang M., Gu Q. (2018). Effect of plasmonic near field on the emittance of plasmon-enhanced photocathode. Nucl. Instrum. Methods Phys. Res. Sect. A Accel. Spectrom. Detect. Assoc. Equip..

[73] Jiang Z., Gu Q., Li X., Wang E., Gaowei M., Liu W. (2021). Monte Carlo simulations of electron photoemission from plasmon-enhanced bialkali photocathode. Phys. Rev. Accel. Beams.

[74] Niigaki M., Hirohata T., Akahori W., Kan H. (2010). Novel field-assisted photocathodes with nanoscale grating antennas. J. Vac. Sci. Technol. B.

[75] Hirohata T., Niigaki M., Mochizuki T., Fujiwara H., Kan H. (2007). Near-infrared photocathode using surface plasmon resonance. Jpn. J. Appl. Phys..

[76] Polyakov A., Senft C., Thompson K.F., Feng J., Cabrini S., Schuck P.J., Padmore H., Peppernick S.J., Hess W.P. (2013). Plasmon-enhanced photocathode for high brightness and high repetition rate X-ray sources. Phys. Rev. Lett..

[77] Pavlenko V., Liu F., Hoffbauer M.A., Moody N.A., Batista E.R. (2016). Kinetics of alkali-based photocathode degradation. AIP Adv..

[78] Li X.-P., Wang J.-Q., Xu J.-Q., Pei S.-L., Xiao O.-Z., He D.-Y., Lv K., Kong X.-C., Peng X.-H. (2017). Constructions and preliminary HV conditioning of a photocathode direct-current electron gun at IHEP. Chin. Phys. Lett..

[79] Zheng L., Du Y., Zhang Z., Qian H., Yan L., Shi J., Zhou Z., Wu X., Su X., Wang D. (2016). Development of S-band photocathode RF guns at Tsinghua University. Nucl. Instrum. Meth. A.

